# Potential causal associations between vitamin D and uric acid: Bidirectional mediation analysis

**DOI:** 10.1038/srep14528

**Published:** 2015-09-29

**Authors:** Ammarin Thakkinstian, Thunyarat Anothaisintawee, Laor Chailurkit, Wipa Ratanachaiwong, Sukit Yamwong, Piyamitr Sritara, Boonsong Ongphiphadhanakul

**Affiliations:** 1 Section for Clinical Epidemiology and Biostatistics, Faculty of Medicine, Ramathibodi Hospital, Mahidol University, Thailand; 2 Department of Family Medicine, Section for Clinical Epidemiology and Biostatistics, Faculty of Medicine, Ramathibodi Hospital, Bangkok, Thailand; 3 Division of Endocrinology, Department of Medicine, Faculty of Medicine, Ramathibodi Hospital, Mahidol University, Thailand; 4 Medical and Health Office, Electricity Generating Authority of Thailand, Nonthaburi, Thailand; 5 Division of Cardiology, Department of Medicine, Faculty of Medicine, Ramathibodi Hospital, Mahidol University, Thailand

## Abstract

Vitamin D deficiency, a major public-health worldwide, is associated with hyperuricemia but casual association is questioned. The study was conducted to determine potential causal associations between 25-hydroxy vitamin D (25(OH)D) and uric acid (UA). A cross-sectional study of the Electricity Generating Authority of Thailand (EGAT3) cohort was conducted. Subjects (n = 2,288) were used to genotype the group-specific component (GC) at *rs2282679* and ATP-binding cassette subfamily G member 2 (ABCG2) at *rs2231142.* Mediation analysis with 1000-replication bootstrap was applied to construct causal pathways i.e., *rs2282679* → 25(OH)D → UA and *rs2231142* → UA → 25(OH)D: The mediator (i.e., 25(OH)D and UA) was firstly regressed on the studied gene (i.e., *rs2282679 and rs2231142*). A potential causal effect of C allele on UA through 25(OH)D was −0.0236 (95% CI: −0.0411, −0.0058), indicating every minor C allele resulted in decreasing the 25(OH)D and then significantly decreased the UA by 0.0236 unit. For the second pathway, the mediation effect was 0.0806 (95% CI: 0.0107, 0.1628); every T allele copy for *rs2231142* increased UA and thus increased 25(OH)D by 0.0806 unit. Our study suggested potential causal associations between the GC gene and UA through the 25(OH)D mediator, and the ABCG2 and the 25(OH)D through the UA mediator but the absolute effects are very clinically small.

Vitamin D deficiency and hyperuricemia are recognized as the major public health concerns worldwide. Prevalence of hyperuricemia has been increasing in both developed and developing countries^1^,^2^. High uric acid (UA) induces urate crystallization in many organs and causes gout, urolithiasis, and acute and chronic nephropathy. In addition, hyperuricemia was also associated with diseases including hypertension^3^,^4^, metabolic syndrome^5^, diabetes mellitus^6^,^7^, and cardiovascular disease^5^,^8^,^9^. Over 1 billion population across the world have been diagnosed with vitamin D insufficiency or deficiency^10^, which caused both skeletal (i.e. rickets^11^ and osteoporosis^12^) and extra-skeletal diseases (i.e. diabetes mellitus^13^, and cardiovascular disease^14^).

Evidences from genome-wide association studies (GWAS) suggested that inherited characteristics play roles in UA and vitamin D metabolism pathways, in which approximately 40% to 60% and 29% to 80% for UA^15^,^16^ and 25-hydroxy vitamin D (25(OH)D) variations^17^,^18^,^19^ could be explained by the genetic background, respectively. About 75% of UA is excreted in proximal tubules of kidneys and the rest is eliminated via the gastrointestinal tract^20^,^21^. Most genes involve in excretion of UA via the urate transporters, which include solute carrier family 2-member (SLC2A9 and SLC22A11), and ATP-binding cassette subfamily G member 2 (ABCG2)^22^,^23^, but the ABCG2 loci were the strongest influence in Asian population^22^. This finding was replicated by individual studies, which found that ABCG2 *rs2231142 C* > *A* increased serum UA concentration^24^ by decreasing the urate transportation rate in proximal tubules^25^.

GWASs also discovered 4 loci which were involved in vitamin D synthesis pathway, in which the group-specific component (GC) at *rs4588* and *rs2282679* and the cytochrome P450 IIR-1 gene (CYP2R1) at *rs10766197* were most significantly associated with serum vitamin D level. This finding was confirmed by individual studies in Asian population^26^,^27^,^28^.

Both vitamin D deficiency and hyperuricemia are associated with the risk of occurrence of chronic diseases, i.e. diabetes mellitus and cardiovascular disease. A number of animal and human studies have suggested that vitamin D and UA metabolism pathways are related. For example, induction of increased circulating UA was found to suppress 1α-hydroxylase leading to lower 1,25(OH)_2_D and increased PTH in rats^29^. Likewise, in humans, administration of allopurinol reduces serum UA with a concurrent increase in 1,25(OH)_2_D and a reduction in PTH^30^,^31^. On the other hand, previous studies in humans suggested negative association between parathyroid hormone (PTH) and serum UA^32^,^33^, which corresponded to findings from a study in postmenopausal women given teriparatide^34^. Low level of vitamin D could leads to hyperuricemia from PTH stimulation. This hypothesis was contradicted by evidences which found positive associations of UA on vitamin D related phenotypes such as bone mineral density (BMD)^35^, dementia^36^, and Parkinson’s disease^37^. It is therefore unclear if vitamin D reciprocally influences UA metabolism and thus constitutes a negative feedback loop commonly found in homeostatic system^38^. Toward this end, we assessed birectional causal pathways of vitamin D and serum UA using a mediation analysis with accounting for GC and ABCG2 polymorphisms.

## Results

A total of 2288 out of 2592 of the EGAT cohort had genotypic data for GC at *rs2282679* and ABCG2 at *rs2231142* SNPs. The mean age and body mass index (BMI) were respectively 39.9 (SD = 6.6) years and 23.9 (SD = 3.8) with male gender was majority (74.3%), see Supplementary Table 1. Lipid profiles were measured with mean cholesterol, triglyceride, HDL, and LDL of 216.7 (SD = 38.8), 129.5 (SD = 89.9), 51.5 (SD = 12.3), and 148.3 (SD = 36.9) mg/dL, respectively. In addition, mean total 25(OH)D and UA were 25.1 (SD = 6.8) ng/mL and 5.6 (SD = 1.5) mg/dL, respectively. Potential causal associations between the 2 SNPs, intermediate phenotypes, and outcomes were assessed as follows:

### GC rs2282679-25(OH)D-UA

**Figure 1:**
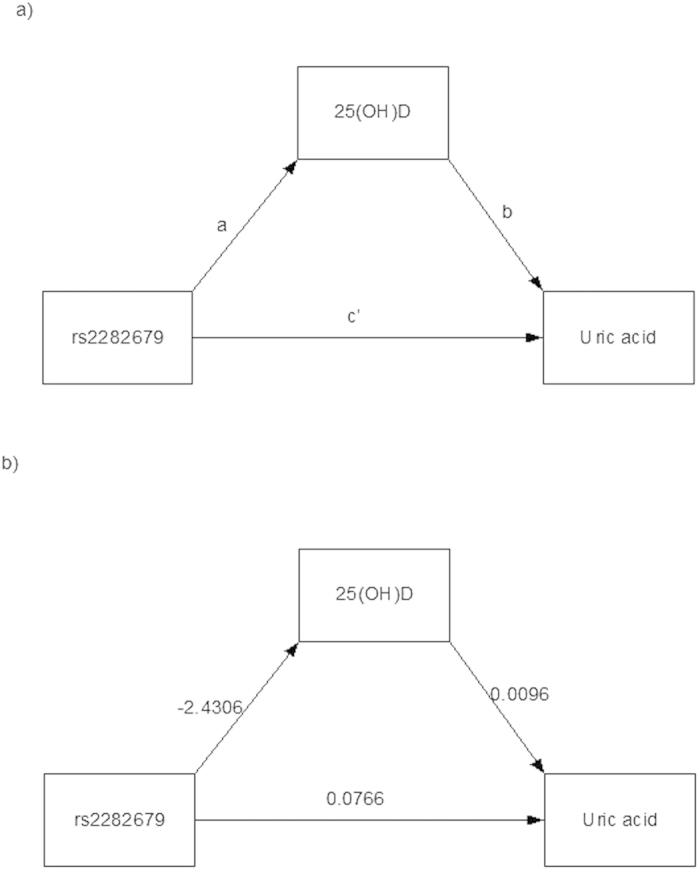
Association between rs2282679, 25(OH)D level, and uric acid; (**a**) A potential causal diagram for path ab and c’ (**b**) Regression coefficients.

Potential causal relationships between *rs2282679*, 25(OH)D, and UA were assessed following a causal diagram in Fig. 1a. Two equations (i.e., *rs2282679* → 25(OH)D and 25(OH)D → UA) were constructed with adjustment for covariables, see Table 1. The results suggested that every one minor C allele in the *rs2282679* → 25(OH)D path would significantly decrease 25(OH)D by 2.4306 (95% confidence interval (CI): −2.8512, −2.0101) ng/mL, see Fig. 1b. The 25(OH)D → UA path suggested that increasing one unit of 25(OH)D would significantly increase UA by 0.0097 (95% CI: 0.0025, 0.0169) mg/dL see Fig. 1b. A bootstrap with 1000 replications yielded the potential causal effect of C allele on UA mediated through 25(OH)D by −0.0236 (95% CI: −0.0411, −0.0058), see Table 2. This could be interpreted that every minor C allele would result in decreased 25(OH)D level and then significantly decreased UA by 0.0236 unit. The minor allele C was also directly associated with UA but this was non-significant after correction for bias (coefficient = 0.0766, 95% CI: −0.0004, 0.1474). The percentage of gene effect contributed by mediation effect (a1b1 path) was 27.9% (95% CI: 24.9%, 30.9%).

### ABCG2 *rs2231142* → UA → 25(OH)D

**Figure 2:**
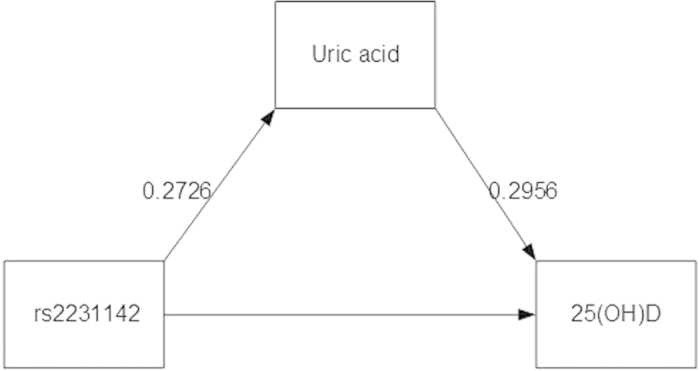
Coefficients of association between rs2231142, uric acid, and 25(OH)D.

The 2 equations (*rs2231142* → UA and UA → 25(OH)D) were constructed with adjusted covariables as displayed in Fig. 2 and Table 3. For the first path, every T allele of *rs2231142* would significantly increase UA by 0.2726 (95% CI: 0.2020, 0.3431) mg/dL. The UA was also significantly correlated with 25(OH)D in the second path, i.e. one unit of UA increase would significantly increase 25(OH)D by 0.2956 (95% CI: 0.0521, 0.5392) ng/mL. A 1000-replication bootstrap suggested the mediation effect of 0.0806 (95% CI: 0.0107, 0.1628), from which could be interpreted that every one copy of minor T allele would increase UA and thus increase 25(OH)D level by 0.0806 unit, see Table 4. The percentage of gene effect contributed through UA mediator (a1b1 path) was 29.5% (95% CI: 28.2%, 30.8%). This minor T allele was also directly associated with 25(OH)D, but this was not significant (coefficient = −0.2471, 95% CI: −0.6371, 0.1606).

#### Sensitivity analysis

A robustness of the results was explored if sequential ignorability (SI) assumptions were violated, see Supplementary Table 2. The proportions of original variances 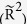 that could be explained if we took into account for invalid SI assumptions were 0.0014 and 0.0012 for casual pathways 1 and 2, respectively. In addition, the unexplained variances that might be explained by unobserved confounders were 0.003 and 0.0025 for these corresponding pathways, indicating very mild effects if the assumptions were violated.

## Discussion

Previous evidences have shown a reverse association between vitamin D status and serum UA in various situation including postmenopausal women^30^, patients with diabetes^31^, or stable renal failure^39^. The causal role of vitamin D in this regard is still unclear. *In vivo*, hyperuricemia has been shown to suppress 1-α hydroxylase and hence lower 1,25(OH)2D with subsequent activation of parathyroid glands. This is in keeping with findings in humans which demonstrated the increased odds of hyperparathyroidism by hyperuricemia^29^. In the present study, we further delineated the interrelationship between vitamin D level and serum UA by determining the potential causal associations of GC *rs2282679* → 25(OH)D → UA and ABCG2 *rs2231142* → UA → 25(OH)D pathways. The findings suggested a potential causal effect of 25(OH)D on UA, in which decreasing vitamin D level from carrying minor allele C would result in decreasing serum UA. In addition, a ABCG2 *rs2231142* → UA → 25(OH)D pathway indicated increasing UA from carrying a minor T allele would lead to increase vitamin D level.

It has been found that hyperuricemia, gout and primary hyperparathyroidism are associated^40^,^41^,^42^. Moreover, the causal role of parathyroid hormone (PTH) in causing hyperuricemia is suggested by the increase in serum uric levels in patients treated with teriparatide^34^. Nevertheless, the underlying mechanism of the observation is unclear. It is likely that PTH possesses a direct effect on UA metabolism, but it is also conceivable that PTH may influence UA through other mediators. Patients with primary hyperparathyroidism are at increased risk of vitamin deficiency^43^ and our finding of the likely causal effect of 25(OH)D on UA suggested vitamin D status as one of the mediators. However, our finding indicated a positive association between vitamin D status and UA which is opposite to the occurrence of lower vitamin D status and hyperuricemia in primary hyperparathyroidism or teriparatide treatment. Clinical trials to investigate the effect of vitamin D supplementation on serum UA are therefore necessary and monitoring of serum UA after vitamin D supplementation to avoid hyperuricemia may be warranted. With regard to the negative causal influence of UA on 25(OH)D suggested by our analyses, studies have shown an inversed association between serum UA and 1,25(OH)2D^30^,^31^. Moreover, administering allopurinol to lower serum UA in patients with gout resulted in an increase in 1,25(OH)2D. However, no change in 25(OH)D or PTH were demonstrated^44^. It therefore still remains to be determined if UA directly affects 25(OH)D as suggested by our study.

High level of UA, the end product of purine metabolism, is known as a cause of lowering kidney function^41^ and gouty arthritis^40^,^42^. Increasing UA level may also induce endothelial dysfunction and thus increase risk to develop diseases such as cardiovascular disease^45^,^46^,^47^, or insulin resistance^48^. Contrastingly, with its antioxidant property, high level of UA was found to increase BMD for all sites and thus decreased the odds of fractures^35^, decreased risk of dementia^36^ and Parkinson’s disease^37^. This might be explained in that UA itself directly affected these clinical outcomes, or its effect was mediated by other intermediate phenotype. A proper cohort studies (i.e., free from clinical endpoint at baseline, measure UA prior to intermediate phenotype/s, and measure the intermediate phenotype/s prior to the interested outcome) are still required to assess a causal effects of UA on clinical endpoints.

Mendelian randomization approach using instrumental variable analysis has been used to assess a causal association pathway between gene and outcome through an intermediate phenotype^49^,^50^. This approach could be not applied to our data because the *rs2282679* polymorphism itself was also directly associated with UA. This was consistent with a finding by Davies *et al.*^51^, in which the studied gene was independently associated with survival in melanoma patients.

Our study has some strengths. We have mapped two causal association of vitamin D and UA pathways using the data from EGAT cohort. A mediation analysis was applied to determine mediation effects of vitamin D on UA and vice versa. This method, also known as process analysis, is the only one of a few statistical methods that have been used to determine a potential causal mechanism or process of how one variable affects the outcome^52^. However, the mediation analysis requires a few important SI assumptions to yield valid results as follows^53^,^54^. First, the studied gene should be ignorable from the outcome and mediator given observed and unobserved confounding factors. Second, the observed mediators (i.e., 25(OH)D for path 1 and UA for path 2) should be independent (i.e. ignorable) from the outcome, given the gene status, pre-observed, and unobserved confounding factors. The two SI assumptions could not be checked directly, but performing a sensitivity analysis would lead to estimate unexplained variance that may be explained by unobserved confounders; which were very low, i.e., 0.3% and 0.25% for pathway 1 and pathway 2. In addition, the first SI assumption should be able to be met because the two studied polymorphisms were randomly allocated since conception as for the Mendelian randomization approach^49^. As a result, violation of the SI assumption should have less effect on our mediation models.

However, our study has some weak points. First, our outcomes of interest are still surrogate or intermediate outcomes, so more clinical endpoints should be followed and assessed. Time of measurements for these intermediate variable/s and end outcomes should be well planned, i.e., the intermediate variables should be measured prior to the occurrence of the end outcome. There may be some other genetic instruments for vitamin D and UA but we had focused only on GC *rs2282679* for vitamin D^55^ and ABCG2 *rs2231142* for UA^56^. Considering those genes together as allelic score, either for instrumental variable or mediation analysis, should be better in explaining outcomes than considering only one polymorphism^57^. Finally, some other important confounders (e.g., dietary intake, sun exposure, vitamin D supplement, etc.) were not considered in our analysis because data were not available. This should be kept in mind that our potential causal effects might be confounded by these unobserved confounders, although the results from sensitivity analysis showed small effects.

In summary, our evidence has suggested a causal associations between GC*(rs2282679)* → 25(OH)D → UA and ABCG2(*rs2231142)* → UA → 25(OH)D pathways, but the effects are very clinically small. The genes contributed approximately 27.9% and 29.5% of total effects on UA and 25(OH)D, respectively. Further cohorts with long term follow up for clinical endpoints and sequential measurement of mediators should be conducted to confirm these findings.

## Methods

This cross-sectional study was baseline data of the Electricity Generating Authority of Thailand (EGAT 3) cohort^58^. Subjects aged 24–54 regardless their disease status were recruited from the headquarters of EGAT in the Bangkok metropolitan area in the year 2009. The extended cohort was aimed to determine genetic factors and markers which were associated with metabolic disorders and bone health. Data collection was performed using a self-administered questionnaire, physical examination, electrocardiography, chest radiography and blood tests. The study was approved by the Institutional Review Board of the Faculty of Medicine at Ramathibodi Hospital, and it was carried out in accordance with the approved guidelines. Written informed consents were obtained from every subject.

### Serum 25-hydroxyvitamin D (25(OH)D) measurement

Serum 25(OH)D_2_ and 25(OH)D_3_ were analyzed by LC-MS/MS with an Agilent 1200 Infinity liquid chromatograph (Agilent Technologies, Waldbronn, Germany) coupled to a QTRAP^®^ 5500 tandem mass spectrometer (AB SCIEX, Foster City CA, USA) using a MassChrom^®^ 25-OH-Vitamin D_3_/D_2_ diagnostics kit (ChromSystems, Munich, Germany). The 25(OH)D assay was performed according to the manufacturer’s instructions. This method used a deuterated 25(OH)D3 as an internal standard to correct for sample and instrument variability. Samples were analyzed using an atmospheric pressure chemical ionization source for maximum sensitivity. The 25(OH)D separation was performed using Chromsystems precipitation reagent and trap column in conjunction with Agilent 1200 HPLC system configured for on-line sample preparation according to the configuration included in the documentation with this method. Briefly, 25(OH)D3 and 25(OH)D2 were extracted by mixing 100 μl of serum sample with 25 μl precipitation reagent and 200 μl of the internal standard solution. The mixture was vortexed for 20 seconds and incubated for 10 minutes at 4 °C. After the mixture was centrifuged for 5 minutes at 9,000 g, the upper layer was transferred to an autosampler vial and 5 μl was injected to the LC-MS/MS. The summation of serum 25(OH)D_2_ and 25(OH)D_3_ was used to reflect vitamin D status. The inter-assay and intra-assay coefficients of variation of total serum 25(OH)D level were 6.3% and 5.0%, respectively.

### Uric acid measurement

Serum uric acid levels were determined using uricase method (Siemens Healthcare Diagnostics Inc., Newark DE, USA). The assay range was 0–20 mg/dl with reference ranges of 2.6–6.0 mg/dl and 3.5–7.2 mg/dl for females and males, respectively. The intra- and inter-assay coefficients of variation were respectively 1.4% and 1.4% at uric acid level 5.1 mg/dl; 1.2% and 1.3% at uric acid level 9.0 mg/dl.

### Genotyping

A standard phenol-chloroform method was used to extract genomic DNA from peripheral bloodleukocytes. The *GC rs2282679* (OMIM: 139200) *and ABCG2 rs2231142* (OMIM: 603756) polymorphisms were genotyped using a TaqMan^®^ assay with allele-specific probes on the ABIPrism 7500 Real-Time PCR system (Applied Biosystems, Foster City, CA). The genotyping call rate was higher than 99%. When the single-nucleotide polymorphism calling was in doubt, direct sequencing was used to provide the correct genotypes.

### Statistical analysis

Data were described using mean and frequency for continuous and categorical data, respectively. Genotyping frequencies for *rs2282679* and *rs2231142* polymorphisms were checked whether their distributions complied with Hardy–Weinberg Equilibrium using an exact test.

Mediation analysis for continuous data^54^,^59^ was applied by constructing two causal pathways, i.e., *rs2282679* → 25(OH)D → UA (see Fig. 1a) and *rs2231142* → UA → 25(OH)D (see Fig. 2). For the former pathway, *rs2282679* was fitted as independent variable, 25(OH)D was mediator, and UA was the outcome of interest. For the later pathway, *rs2231142* was the independent variable, UA was the mediator, and 25(OH)D was the outcome of interest. Causal equations for both pathways were constructed as follows: The mediator for each pathway was firstly regressed on the studied gene (called path a_1_, see Fig. 1a). The outcome variable was then regressed on mediator and studied gene (path b_1_). The 2 studied genes were fitted in the equations as additive effects by assigning 0, 1 and 2 for major homozygous, heterozygous, and minor homozygous genotypes, respectively. The equations for these paths are as follows:


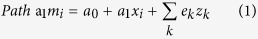



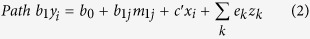


*where* x_i_ = 0, 1, 2 for major homozygous, heterozygous, and minor homozygous genotypes; m = 25(OH)D and UA for pathway 1 and 2, respectively; z_k_ = confounders.

Confounders including age, gender, BMI, and triglyceride were included in the two pathways. Triglyceride was considered instead of other lipid profiles because this variable was the most significantly associated with mediator and outcome, and to avoid colinearity among them if they were included in the same model. A potential causal mediation effect was then estimated using the product-of-coefficient method, i.e., a_1_b_1_^52^,^60^,^61^. A bootstrap analysis with 1,000 replications was then applied to estimate average causal mediation effects without requiring the assumption of normality^59^,^62^. For each bootstrap, the causal mediation effect was estimated, averaged across 1000 replications, and its corresponding 95% CI was then determined using bias-corrected bootstrap technique.

A sensitivity analysis was performed to determine robustness of effects if the SI assumptions were violated^63^. The proportion of unexplained variances 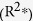 that were explained by unobserved confounders, and the proportions of original variances 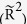 which were explained by unobserved confounders in the mediator and outcome models were then estimated. Analyses were performed using STATA 13.0 software. STATA commands used for all analyses were provided in the Supplement document. A *P*-value < 0.05 was considered statistically significant.

## Additional Information

**How to cite this article**: Thakkinstian, A. *et al.* Potential causal associations between vitamin D and uric acid: Bidirectional mediation analysis. *Sci. Rep.* **5**, 14528; doi: 10.1038/srep14528 (2015).

## Supplementary Material

Supplementary Information

Supplementary Information

## Figures and Tables

**Table 1:** Mediation analysis of *rs2282679,* 25(OH)D level, and uric acid.

Equation	Factors	b	SE	t	P	95% CI	
						LL	UL
25(OH)D	*rs2282679*	−2.4306	0.2146	−11.328	<0.001	−2.8512	−2.0101
	Age	0.1226	0.0192	6.385	<0.001	0.0850	0.1602
	Gender	−4.4174	0.3178	−13.899	<0.001	−5.0403	−3.7945
	BMI	−0.1105	0.0385	−2.870	0.004	−0.1859	−0.0350
	Triglyceride	0.0068	0.0016	4.236	<0.001	0.0037	0.0100
UA	25(OH)D	0.0097	0.0037	2.625	0.009	0.0025	0.0169
	*rs2282679*	0.0765	0.0387	1.979	0.048	0.0007	0.1524
	Age	−0.0028	0.0034	−0.815	0.415	−0.0094	0.0039
	Gender	−1.6602	0.0581	−28.592	<0.001	−1.7740	−1.5464
	BMI	0.0843	0.0068	12.471	<0.001	0.0711	0.0976
	Triglyceride	0.0024	0.0003	8.515	<0.001	0.0019	0.0030

*rs2282679* was fitted as additive effect with 0,1, and 2 for AA, AC, and CC, respectively.

b, coefficient; BMI, body mass index; CI, confidence interval; LL, lower limit of coefficient; P, p value; SE, standard error; t, t-test; UA, uric acid.

**Table 2:** Causal effects of *rs2282679* on uric acid that was mediated by total 25(OH)D.

Effects	Pathway	b	SE	Z	P	Bias	95% CI	
							LL	UL
Indirect	*GC* → 25(OH)D → UA (a_1_b_1_)	−0.0235	0.0093	−2.521	0.012	0.0004	−0.0411	−0.0058
Direct	*GC* → UA (c’)	0.0765	0.0384	1.994	0.046	0.0018	−0.0004	0.1474

GC at *rs2282679*.

b, coefficient; BMI, body mass index; CI, confidence interval; LL, lower limit of coefficient; P, p value; SE, standard error; UA, uric acid; Z, Z-test.

**Table 3:** Mediation analysis of *rs2231142* effect on UA through 25(OH)D.

Equations	Factors	b	SE	t	P	95% CI	
						LL	UL
UA	ABCG	0.2726	0.0360	7.575	<0.001	0.2020	0.3431
	Age	−0.0015	0.0033	−0.436	0.663	−0.0080	0.0051
	Gender	−1.6895	0.0552	−30.625	<0.001	−1.7977	−1.5814
	BMI	0.0824	0.0067	12.334	<0.001	0.0693	0.0955
	Triglyceride	0.0025	0.0003	8.799	<0.001	0.0019	0.0030
25(OH)D	UA	0.2956	0.1243	2.379	0.017	0.0521	0.5392
	ABCG	−0.2471	0.2155	−1.147	0.251	−0.6695	0.1752
	Age	0.1177	0.0197	5.972	<0.001	0.0791	0.1563
	Gender	−3.8216	0.3880	−9.849	<0.001	−4.5821	−3.0610
	BMI	−0.1258	0.0408	−3.083	0.002	−0.2058	−0.0458
	Triglyceride	0.0062	0.0017	3.707	<0.001	0.0029	0.0095

*rs2231142* was fitted as additive effect with 0, 1, and 2 for GG, GT, and TT, respectively.

b, coefficient; BMI, body mass index; CI, confidence interval; LL, lower limit of coefficient; P, p value; SE, standard error; t, t-test; UA, uric acid.

**Table 4:** Causal effects of *rs2231142* on 25(OH)D through uric acid.

Effects	Pathway	b	SE	Z	P	Bias	95% CI	
							LL	UL
Indirect	*ABCG* → UA → 25(OH)D (a_1_b_1_)	0.0806	0.0385	−2.090	0.037	0.0013	0.0106	0.1619
Direct	*ABCG* → 25(OH)D (c’)	−0.2471	0.2022	−1.222	0.222	0.0017	−0.6371	0.1606

ABCG at *rs2231142*.

b, coefficient; BMI, body mass index; LL, lower limit of coefficient; P, p value; SE, standard error; UA, uric acid; Z, Z-test.
