# Supplementary material for: Potential causal associations between vitamin D and uric acid: Bidirectional mediation analysis
Source: Sci Rep. 2015 Sep 29;5:14528. doi: 10.1038/srep14528 (PMC4586492; doi:10.1038/srep14528)
Supplement: Supplementary Information [file srep14528-s1.pdf]

Potential casual associations between vitamin D and uric acid: Bidirectional mediation analysis

Ammarin Thakkinstian

Section for Clinical Epidemiology and Biostatistics, Faculty of Medicine, Ramathibodi Hospital, Mahidol University, Thailand.

e-mail: ammarin.tha@mahidol.ac.th

Thunyarat Anothaisintawee

Department of Family Medicine, Section for Clinical Epidemiology and Biostatistics, Faculty of Medicine, Ramathibodi Hospital, Bangkok, Thailand

e-mail: thunyarat.ano@mahidol.ac.th

Suwanne Chanprasertyothin

Office of Research Academic Affairs and Innovation, Faculty of Medicine, Ramathibodi Hospital, Mahidol University, Bangkok, Thailand

e-mail: suwannee.cha@mahidol.ac.th

Laor Chailurkit

Division of Endocrinology, Department of Medicine, Faculty of Medicine, Ramathibodi Hospital, Mahidol University, Thailand

e-mail: laor.cha@mahidol.ac.th

Wipa Ratanachaiwong

Medical and Health Office, Electricity Generating Authority of Thailand, Nonthaburi, Thailand

e-mail: wipa.r@egat.co.th

Sukit Yamwong

Division of Cardiology, Department of Medicine, Faculty of Medicine, Ramathibodi Hospital, Mahidol University, Thailand

e-mail: sukit.yam@mahidol.ac.th

Piyamitr Sritara

Division of Cardiology, Department of Medicine, Faculty of Medicine, Ramathibodi Hospital, Mahidol University, Thailand

e-mail: pyamitr.sri@mahidol.ac.th

Boonsong Ongphiphadhanakul

Division of Endocrinology, Department of Medicine, Faculty of Medicine, Ramathibodi Hospital, Mahidol University, Thailand

e-mail: boonsong.ong@mahidol.ac.th

Statistical methods for mediation analysis

Mediation analysis of *rs2282679*, 25(OH)D level, and uric acid

```
Independent variable (iv) = gc2

Mediator (m) = totald

Dependent variable (dv) = uric

Covariables (cv)  = age gender bmi1dom trig


  sureg  (totald gc2 age gender bmi1 trig   ) (uric totald gc2 age gender bmi1 trig  )

  Seemingly unrelated regression

----------------------------------------------------------------------

Equation          Obs  Parms        RMSE    "R-sq"       chi2        P

----------------------------------------------------------------------

totald           2266      5    6.236256    0.1594     429.62   0.0000

uric             2266      6    1.093692    0.4465    1827.77   0.0000

----------------------------------------------------------------------


------------------------------------------------------------------------------

             |      Coef.   Std. Err.      z    P>|z|     [95% Conf. Interval]

-------------+----------------------------------------------------------------

totald       |

         gc2 |  -2.430616   .2145764   -11.33   0.000    -2.851178   -2.010054

         age |   .1226062    .019202     6.39   0.000      .084971    .1602415

      gender |  -4.417423   .3178173   -13.90   0.000    -5.040333   -3.794512

     bmi1dom |  -.1104589   .0384917    -2.87   0.004    -.1859012   -.0350166

        trig |   .0068283    .001612     4.24   0.000     .0036687    .0099878

       _cons |   31.01102   1.317632    23.54   0.000     28.42851    33.59353

-------------+----------------------------------------------------------------

uric         |

      totald |   .0096727   .0036842     2.63   0.009     .0024518    .0168935

         gc2 |   .0765492   .0386824     1.98   0.048     .0007331    .1523654

         age |  -.0027685   .0033977    -0.81   0.415     -.009428    .0038909

      gender |  -1.660172    .058065   -28.59   0.000    -1.773977   -1.546366

     bmi1dom |     .08434   .0067628    12.47   0.000     .0710852    .0975949

        trig |   .0024168   .0002838     8.51   0.000     .0018605    .0029731

       _cons |   5.088833   .2577825    19.74   0.000     4.583588    5.594077

------------------------------------------------------------------------------
```

Performing bootstrap

```
program drop bootmed1

       program bootmed1, rclass

  syntax [if] [in]

  sureg  (totald gc2 age gender bmi1 trig   ) (uric totald gc2 age gender bmi1 trig  )

  return scalar a1b1  = [totald]_b[gc2]*[uric]_b[totald]

  return scalar c1 = [uric]_b[gc2]

  return scalar total = abs([totald]_b[gc2]*[uric]_b[totald]) + ///

                    [uric]_b[gc2]

       end


bootstrap r(a1b1)  r(c1) r(total), reps(1000) saving(gc2_totald_uric_med_bs_29_04_2014,
replace) nodots: bootmed1

estat boot, percentile bc
```

Sensitivity analysis

```
*a1b1:


medsens (regress totald gc2 age gender bmi1 trig  )  (regress uric totald gc2 age gender bmi1
trig), mediate(totald) treat(gc2) sims(1000) graph
```

Mediation analysis for rs2311142-uric-totald

```
iv = abcg2

m = uric

dv = totald

sureg  (uric abcg2 age gender bmi1 trig   ) (totald uric abcg2 age gender bmi1 trig  )
```

Bootstrap

```
program drop bootmed2

      program bootmed2, rclass

  syntax [if] [in]

  sureg  (uric abcg2 age gender bmi1 trig   ) (totald uric abcg2 age gender bmi1 trig  )

  return scalar a1b1  = [uric]_b[abcg2]*[totald]_b[uric]

  return scalar c1 = [totald]_b[abcg2]

  return scalar total = abs([uric]_b[abcg2]*[totald]_b[uric]) + ///

                  abs([totald]_b[abcg2])

      end


bootstrap r(a1b1)  r(c1) r(total), reps(1000) saving(abcg2_uric_totald_med_bs_30_04_2014,
replace) nodots: bootmed2
```

Sensitivity analysis

```
*a1b1 for abcg2-uric-totald


medsens (regress uric abcg2 age gender bmi1 trig  )  (regress totald uric abcg2 age gender
bmi1 trig), mediate(uric) treat(abcg2) sims(1000) graph
```
