# Supplementary material for: Potential causal associations between vitamin D and uric acid: Bidirectional mediation analysis
Source: Sci Rep. 2015 Sep 29;5:14528. doi: 10.1038/srep14528 (PMC4586492; doi:10.1038/srep14528)
Supplement: Supplementary Information [file srep14528-s2.pdf]

**Potential causal associations between vitamin D and uric acid: Bidirectional mediation analysis**

Ammarin Thakkinstian

Section for Clinical Epidemiology and Biostatistics, Faculty of Medicine, Ramathibodi Hospital, Mahidol University, Thailand.

e-mail: ammarin.tha@mahidol.ac.th

Thunyarat Anothaisintawee

Department of Family Medicine, Section for Clinical Epidemiology and Biostatistics, Faculty of Medicine, Ramathibodi Hospital, Bangkok, Thailand

e-mail: thunyarat.ano@mahidol.ac.th

Suwanne Chanprasertyothin

Office of Research Academic Affairs and Innovation, Faculty of Medicine, Ramathibodi Hospital, Mahidol University, Bangkok, Thailand

e-mail: suwannee.cha@mahidol.ac.th

Laor Chailurkit

Division of Endocrinology, Department of Medicine, Faculty of Medicine, Ramathibodi Hospital, Mahidol University, Thailand

e-mail: laor.cha@mahidol.ac.th

Wipa Ratanachaiwong

Medical and Health Office, Electricity Generating Authority of Thailand, Nonthaburi, Thailand

e-mail: wipa.r@egat.co.th

Sukit Yamwong

Division of Cardiology, Department of Medicine, Faculty of Medicine, Ramathibodi Hospital, Mahidol University, Thailand

e-mail: sukit.yam@mahidol.ac.th

Piyamitr Sritara

Division of Cardiology, Department of Medicine, Faculty of Medicine, Ramathibodi Hospital, Mahidol University, Thailand

e-mail: pyamitr.sri@mahidol.ac.th

Boonsong Ongphiphadhanakul

Division of Endocrinology, Department of Medicine, Faculty of Medicine, Ramathibodi Hospital, Mahidol University, Thailand

e-mail: boonsong.ong@mahidol.ac.th

Supplement Table 1. Characteristics of subjects

| Characteristics | Mean | SD |
|---|---|---|
| Age, years | 39.9 | 6.6 |
| BMI,kg/$m^2$ | 23.9 | 3.8 |
| Total cholesterol, ,mg/dL | 216.7 | 38.8 |
| Triglyceride, mg/dL | 129.5 | 89.9 |
| HDL, mg/dL | 51.5 | 12.3 |
| LDL,mg/dL | 148.3 | 36.9 |
| 25(OH)D, ng/mL | 25.1 | 6.8 |
| UA, mg/dL | 5.6 | 1.5 |
| **Characteristics** | **Number** | **%** |
| Gender | | |
| Male | 1,701 | 74.3 |
| Female | 587 | 25.7 |
| *rs2282679* | | |
| *AA* | 1,314 | 57.4 |
| *AC* | 833 | 36.4 |
| *CC* | 141 | 6.2 |
| *rs2311142* | | |
| *GG* | 1231 | 53.8 |
| *GT* | 884 | 38.6 |
| *TT* | 173 | 7.6 |

BMI, body mass index; UA, uric acid

Supplement Table 2. Sensitivity analysis

| Outcome | σ at which ACME=0 | $R^{2*}$ | $\tilde{R}^2$ |
|---|---|---|---|
| UA | 0.0551 | 0.0030 | 0.0014 |
| 25(OH)D | 0.0499 | 0.0025 | 0.0012 |

UA, uric acid

$R^{2*}$, proportion of unexplained variances

$\tilde{R}^2$, proportion of original variances
